# Identification and characterization of the vertebrate motile ciliome

**DOI:** 10.1186/2046-2530-1-S1-O10

**Published:** 2012-11-16

**Authors:** S Roy, S Choksi, D Babu, X Yu, D Lau, HK Ho, KN Wong, P Panse, L Hao, S Vij, S Chen

**Affiliations:** 1 Institute of Molecular and Cell Biology, Singapore

## 

Our previous work with the zebrafish embryo had uncovered a master regulatory role for the winged-helix transcription factor FoxJ1 in motile ciliogenesis. In humans, dysfunction of motile cilia leads to Primary Ciliary Dyskinesia (PCD). Mutations in only a few genes have thus far been associated with PCD. To identify new genes that are required for motile cilia formation and function, we performed a genome-wide screen to identify the targets of FoxJ1. Approximately 650 genes with mammalian orthologs were found to be up-regulated in response to FoxJ1 over-exopression in zebrafish embryos. Remarkably, all of the genes that have yet been linked with PCD figure on our list of FoxJ1 targets, implying that many of the new genes that we have identified could be mutated in the uncharacterized cases of PCD. To determine how the novel FoxJ1 targets affect cilia, we have carried out systematic gene knock-down using morpholinos, and assayed for perturbations in ciliary architecture and the developmental and physiological read-outs of defective ciliary activity. In addition, using GFP-tagged versions of the proteins, we have performed localization studies with respect to the ciliary apparatus, and employed the yeast 2 hybrid assay to identify interacting partners. Finally, with chromatin immunoprecipitation (ChIP) we have surveyed for FoxJ1 binding sites throughout the zebrafish genome. Our findings indicate that the screen has successfully identified a very large number of novel genes required for ciliary development and function. We believe that these novel ciliary genes will help us to better understand the causes of human ciliopathies.

http://www.imcb.a-star.edu.sg/php/sudipto.php

